# Crystallization and precipitation of phosphate from swine wastewater by magnesium metal corrosion

**DOI:** 10.1038/srep16601

**Published:** 2015-11-12

**Authors:** Haiming Huang, Jiahui Liu, Yang Jiang

**Affiliations:** 1 Hebei Key Laboratory of Applied Chemistry, School of Environmental and Chemical Engineering, Yanshan University, Qinhuangdao 066004, PR China

## Abstract

This paper presents a unique approach for magnesium dosage in struvite precipitation by Mg metal corrosion. The experimental results showed that using an air bubbling column filled with Mg metal and graphite pellets for the magnesium dosage was the optimal operation mode, which could significantly accelerate the corrosion of the Mg metal pellets due to the presence of graphite granules. The reaction mechanism experiments revealed that the solution pH could be used as the indicator for struvite crystallization by the process. Increases in the Mg metal dosage, mass ratio of graphite and magnesium metal (G:M) and airflow rate could rapidly increase the solution pH. When all three conditions were at 10 g L^–1^, 1:1 and 1 L min^–1^, respectively, the phosphate recovery efficiency reached 97.5%. To achieve a high level of automation for the phosphate recovery process, a continuous-flow reactor immersed with the graphite-magnesium air bubbling column was designed to harvest the phosphate from actual swine wastewater. Under conditions of intermittently supplementing small amounts of Mg metal pellets, approximately 95% of the phosphate could be stably recovered as struvite of 95.8% (±0.5) purity. An economic analysis indicated that the process proposed was technically simple and economically feasible.

It is well known that phosphorus (P) is not only an essential element present in all living organisms, but a major nutrient causing eutrophication in water bodies^1^. At the same time, the P derived from phosphate rock is a non-renewable resource with limited reserves, which becomes progressively depleted due to the huge consumption in the agricultural and industrial production, annually. Some researchers have predicted that the known phosphate rock reserves in the world will be exhausted within 100 years if the current increased depletion rate remains unchanged^2^,^3^. To protect this important non-renewable resource, some of the main phosphate-producing countries such as China and the United States have ranked phosphate rock as a strategic resource, and implemented management of export control^4^. Over the past several years, the international market price of phosphate rock has rapidly risen for various reasons. Therefore, the recovery of phosphate from wastewaters is of great significance in the prevention of eutrophication and for the protection of phosphate rock.

Swine wastewater is a typical type of wastewater containing high phosphate concentrations^5^,^6^. The P in the wastewater is generally present as orthophosphate (P_T_), with great eutrophication potential. To prevent the eutrophication of public water bodies, swine farms are forced to reduce the P levels entering the surface waters and keep within the set water quality legislation standards. For this purpose, various biological and chemical processes such as enhanced biological P removal 7, calcium phosphate (Ca_*x*_(PO_4_)_*y*_, CP)/struvite (MgNH_4_PO_4_ · 6H_2_O) crystallization^8^,^9^,^10^, marine macro algae (*Kappaphycus alvarezii*) biosorption^11^ and activated aluminum oxide adsorption^1^, were investigated. Among these processes, struvite crystallization is recognized as an ideal process for phosphate recovery due to its high reaction rate and P recovery efficiency. Due to the Mg^2+^ deficiency in swine wastewater, MgCl_2_ and MgSO_4_ are often used as the magnesium sources for struvite precipitation. Although a high degree of phosphate removal could be achieved by using these salts as magnesium sources, their dosage and the adjustment of the solution pH were difficult to control well due to fluctuation in the phosphate concentration of the wastewaters; the use of these salts also readily increased the salinity in the effluent, inhibiting the microbial activity in the biological treatment process that followed^12^,^13^. Further, to reduce the cost of phosphate recovery, some low-cost magnesium sources such as low-grade MgO and Mg(OH)_2_ were also utilized as magnesium sources^14^,^15^. Unfortunately, these reagents were found to significantly decrease the purity of the harvested struvite due to the excess added MgO/Mg(OH)_2_.

Over the recent years, struvite crystallization with the use of magnesium sacrificial anode as the source of magnesium has gained interest as a novel route to phosphate recovery. Kruk *et al.*^16^ reported that the use of a high-purity magnesium alloy cast anode was very effective for the recovery of high-quality struvite from water solutions, and could achieve 4 mg PO_4_-P cm^–1^·h^–1^ of the highest P-removal rate, at an electric current density of 45 A m^–2^. Hug and Udert^17^ demonstrated that utilizing a sacrificial magnesium electrode for the magnesium dosage in struvite precipitation was technically simple and economically feasible. It was found that 3.7 mg P cm^–1^·h^–1^ could be achieved at an impressed current density of 55 A m^–2^. Magnesium is a type of metal readily corroded or oxidized in aqueous solutions. Its poor corrosion resistance results from the high intrinsic dissolution tendency of magnesium^18^. Song *et al.*^19^ found that the presence of Cl^–^ ions in the solution could accelerate the corrosion of Mg metal and promote the release of Mg^2+^. As swine wastewater has a high concentration of Cl^–^ ions, providing Mg^2+^ for struvite precipitation by the direct corrosion of the Mg metal may be an interesting process for phosphate recovery from swine wastewater.

In this study, high-purity Mg metal granules with greater specific surface area, compared with the Mg metal plate, was used as the magnesium source of struvite precipitation. The main objective of this study was to investigate the feasibility of recovering the phosphate from swine wastewater using struvite precipitation by the corrosion of Mg metal. First, laboratory-scale experiments were conducted to determine the reaction mechanism and the optimal operation mode of the process proposed. Second, the optimal conditions for struvite crystallization by the corrosion of Mg metal were investigated. Third, based on the results of the experiments mentioned above, a continuous-flow reactor using the proposed struvite precipitation process was designed for phosphate recovery from actual swine wastewater. Finally, an economic analysis and comparison was done.

## Results and Discussion

### Struvite crystallization by magnesium metal corrosion

**Figure 1:**
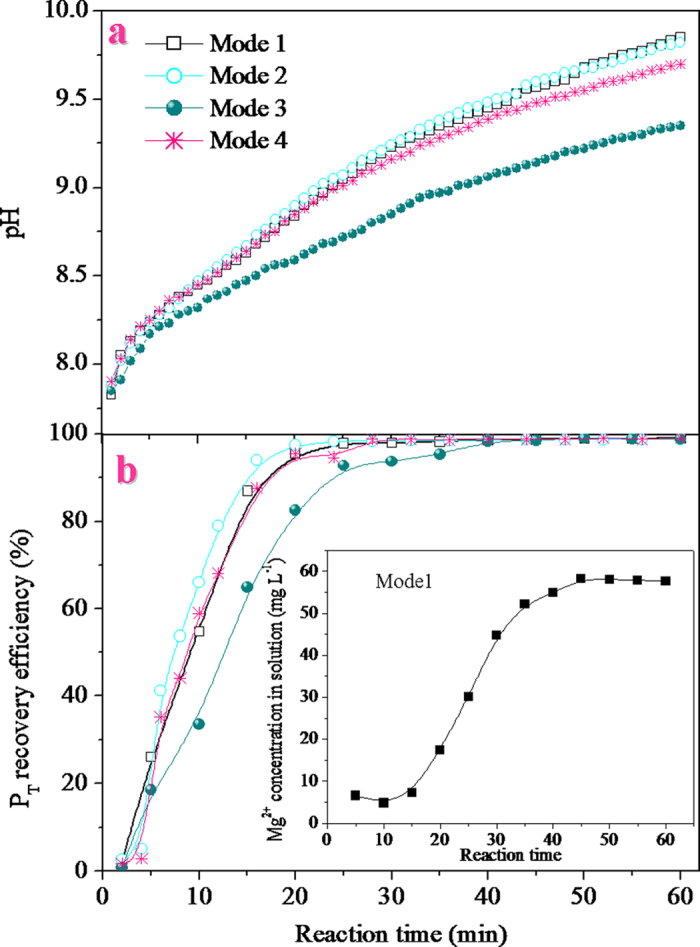
Effects of different operation modes on the solution pH and the P_T_ recovery efficiency (G:M mass ratio, 1:1; airflow rate, 1 L min^–1^; Mg metal dosage, 6 g L^–1^).

The changes in the solution pHs and P_T_ recovery efficiencies for different operation modes (*M*_1_–*M*_4_) with reaction time are shown in Fig. 1, which reveal that the operation modes significantly affect the struvite crystallization by the corrosion of Mg metal. As seen in Fig. 1a, the solution pHs in all the operation modes tested, increased rapidly with an increase in the reaction time. For example, in *M*_4_, the pH of the solution rapidly increased from 7.9 to 9.7 when the reaction time rose from 1 min to 60 min. At an identical reaction time, the solution pHs were at their highest for *M*_1_ and *M*_2_, followed by *M*_4_ and was the least for *M*_3_. As shown in Fig. 1b, the P_T_ recovery efficiencies for all the modes rapidly increased first during 20 min, and then gradually plateaued between 20 min and 60 min. For instance, the P_T_ recovery efficiency in *M*_4_ was observed to rapidly reach 95.4% when the reaction time reached 20 min. With further increase in the reaction time to 30 min, the P_T_ recovery efficiency reached a maximum of 98.8% and then plateaued after 30 min.

**Figure 2:**
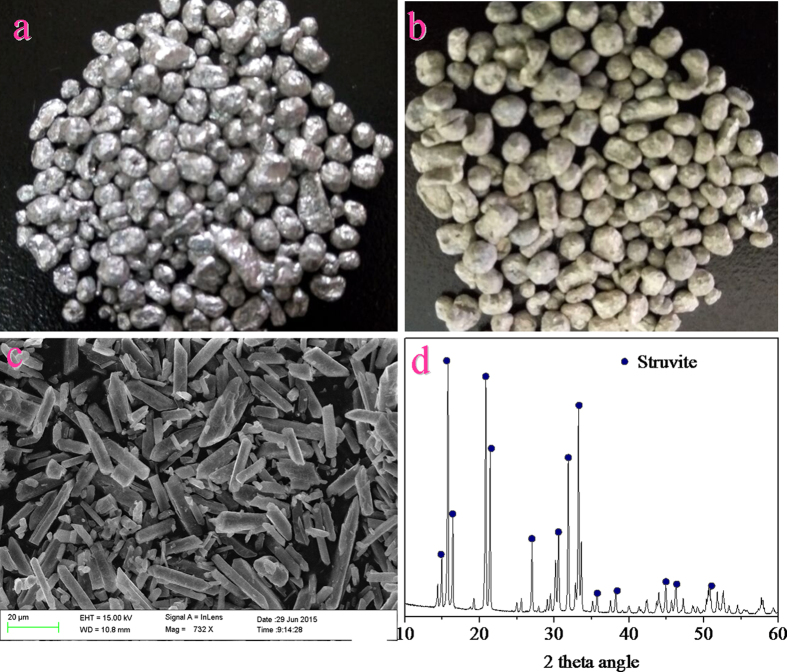
The pictures of the Mg metal before (a) and after (b) experiments, and the SEM micrograph519 (c) and XRD patterns (d) of the collected precipitates.

In this study, the Mg metal corrosion may play a dual function to provide Mg^2+^ for struvite crystallization and to raise the solution pH of the reaction system. Bare Mg metal has a standard reduction potential of –2.37 V_nhe_ at 25 °C^20^, which can result in the hydrogen evolution reaction occurring at high rates on Mg. This concurred with the phenomenon observed during the course of the experiments, during which several air bubbles could be seen forming on the surfaces of the Mg metal pellets. However, in aqueous solutions, the corrosion potential of the Mg metal is actually around −1.7 V_nhe_ 18. This suggests that the surface of bare Mg metal may be covered by a surface film, which blocks the direct contact with the solution and provides some corrosion protection for Mg metal^19^,^20^,^21^. During the course of the experiments, we observed that the unique metallic luster of the Mg metal (Fig. 2a) completely disappeared after corrosion, and its surface was covered by a yellow film of rust (Fig. 2b). Therefore, the Mg metal in aqueous solutions may undergo the following galvanic cell reactions [Eqs (1, 2)]^22^ and chemical reaction [Eq. (3)].


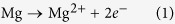



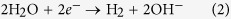



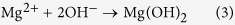


Besides the galvanic cell reactions mentioned above, another anodic partial reaction having two sequential steps may also occur during the corrosion of Mg metal, which involves the formation of uni-positive Mg^+^ as a short-lived intermediate [Eq. (4)]^20^,^23^,^24^ and the conversion of Mg^+^ to the equilibrium Mg^2+^ ion [Eq. (5)].


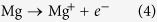



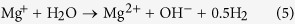


The Mg corrosion reactions may cause a local supersaturation of the Mg^2+^ and OH^–^ at the Mg metal surface, resulting in the formation and growth of the Mg(OH)_2_ film. However, the hydrogen gas produced could denude the film and convert the solid Mg(OH)_2_ into solution. Thus, struvite crystallization in the solution involved the following chemical reactions.


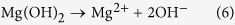



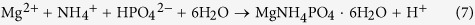


Solution pH and supersaturation are the two important parameters that influence struvite crystallization^25^,^26^. Supersaturation is the state of a solution where the ion activity product of struvite is greater than its solubility product (*K*_sp_)^27^. When the concentrations of the constituted ions (Mg^2+^, NH_4_^+^, and PO_4_^3–^) of struvite are supersaturated, it results in struvite crystallization. As the existing species of the three constituted ions of struvite were significantly influenced by solution pH^28^, the pH of the solution may, in fact, play the most important role in the process of struvite crystallization. In the experiments, changes in the Mg^2+^ concentration of the solution in the first mode were observed with reaction time (Fig. 1b). It was found that the changing profile of the Mg^2+^ concentration was similar to that of the P_T_ recovery efficiency. This suggested that the P_T_ recovery efficiency increased with an increase in the Mg^2+^ concentration in solution. According to the Eq. (6), it can be confirmed that the Mg^2+^ concentration in the solution was closely related to the solution pH. Therefore, the solution pH could be used as a reliable indicator of struvite formation. Based on the results in Fig. 1, it was confirmed that the solution pH ranging from 8.8 to 9 was optimal for the recovery of phosphate from swine wastewater by the corrosion of Mg metal. This finding is consistent with the results reported in earlier literatures^12^,^29^,^30^. Under such conditions, the P_T_ recovery efficiency reached around 96%, and the Mg^2+^ concentration of the solution was lower than 20 mg/L. Although a further increase in the pH could cause a slight increase in the P_T_ recovery efficiency, this would result in excess magnesium loss and a decrease in the struvite purity^31^. The SEM and XRD characterizations of the precipitates collected under optimal conditions indicated that the morphology of the crystalline products with smooth surfaces was regular and needle-shaped (Fig. 2c) and the component was struvite (Fig. 2d).

Song and Atrens^18^ reported that impurity and second phases could be used as the local cathodes to accelerate the local galvanic corrosion of Mg metal. However, according to the results shown in Fig. 1, it can be seen that the presence of the graphite pellets in *M*_2_ did not obviously accelerate the corrosion of the Mg metal compared with *M*_1_. This may be due to the fact that most of the graphite pellets in *M*_2_ were not in contact with the Mg metal because of the rapid stirring. Nevertheless, when *M*_4_ was adopted in the experiments, it was found that the corrosion rate of the Mg metal was obviously greater than that in *M*_3_. This may be attributed to the direct contact of the Mg metal and graphite pellets in the air bubbling column (in *M*_4_). Although the increase rate of pH in *M*_1_ and *M*_2_ exceeded those in the *M*_3_ and *M*_4_, it was difficult to rapidly stop the reaction and to remove the Mg metal from the solution. This could readily incur excessive Mg metal corrosion, resulting in increased magnesium loss. Nevertheless, this problem could be well avoided in the *M*_3_ and *M*_4_ with the air bubbling column, in which the Mg metal corrosion could rapidly be stopped by removing the column out of the solution. Based on the dual considerations of the rate of Mg metal corrosion, and the simplicity of the operation, it was considered that the *M*_4_ mode was the optimal one, and subsequent experiments were conducted in this mode.

### Optimal conditions for struvite crystallization by Mg metal corrosion

#### Effect of magnesium metal dosage

**Figure 3:**
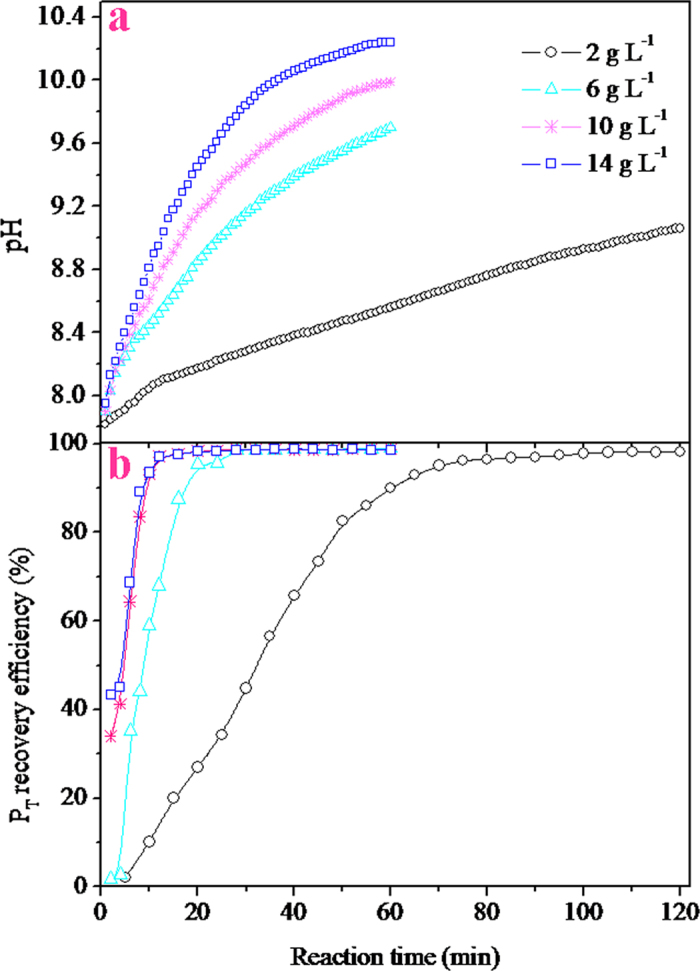
Effect of the dosage of the Mg metal on the solution pH and the P_T_ recovery efficiency (G:M mass ratio, 1:1; airflow rate, 1 L min^–1^).

The results of the experiments to determine the effect of Mg metal dosage on the P_T_ recovery are shown in Fig. 3. It is observed that the dosage of the Mg metal exerts a significant influence on the pH of the solution and the P_T_ recovery efficiency. At a given reaction time, the solution pH increased with the increase in the dosage of the Mg metal (Fig. 3a). For example, when the reaction time was 20 min, the solution pH rapidly increased from 8.17 to 9.45 when the dosage of the Mg metal was increased from 2 g L^–1^ to 14 g L^–1^. This phenomenon may be occurring because under the conditions of maintaining a certain particle size of the Mg metal pellets, increasing its quantity would imply an increase in the surface area of the Mg metal coming into contact with the solution. This could cause the corrosion of more Mg metal pellets, resulting in the rapid increase in the pH of the solution. Therefore, this was also the reason for the corresponding accelerated recovery of P_T_. As shown in Fig. 3b, when the reaction time was at a given value, a rapid increase in the P_T_ recovery efficiency was observed at the dosage of the Mg metal ranging between 2 and 10 g L^–1^; however, further increases in the dosage (>10 g L^–1^) caused no further increase in the P_T_ recovery efficiency. This was attributed to the rapid achievement of the optimal solution pH at the Mg metal dosage in the range of 10 to 14 g L^–1^. When the Mg metal dosage was 10 g L^–1^ and the reaction time 15 min, the solution pH and the P_T_ recovery efficiency were 8.91 and 96.3%, respectively. By comparing these results with those of the published literatures, it was confirmed that the performance of phosphate recovery by the process proposed was completely comparable to those of the magnesium sacrificial anode process reported by Kruk *et al.*^16^ and Hug and Udert^17^. Further, the proposed corrosion process of the Mg metal did not necessitate any additional electrolytic equipment, which could significantly reduce the investment cost of the project.

#### Effect of G:M mass ratio

**Figure 4:**
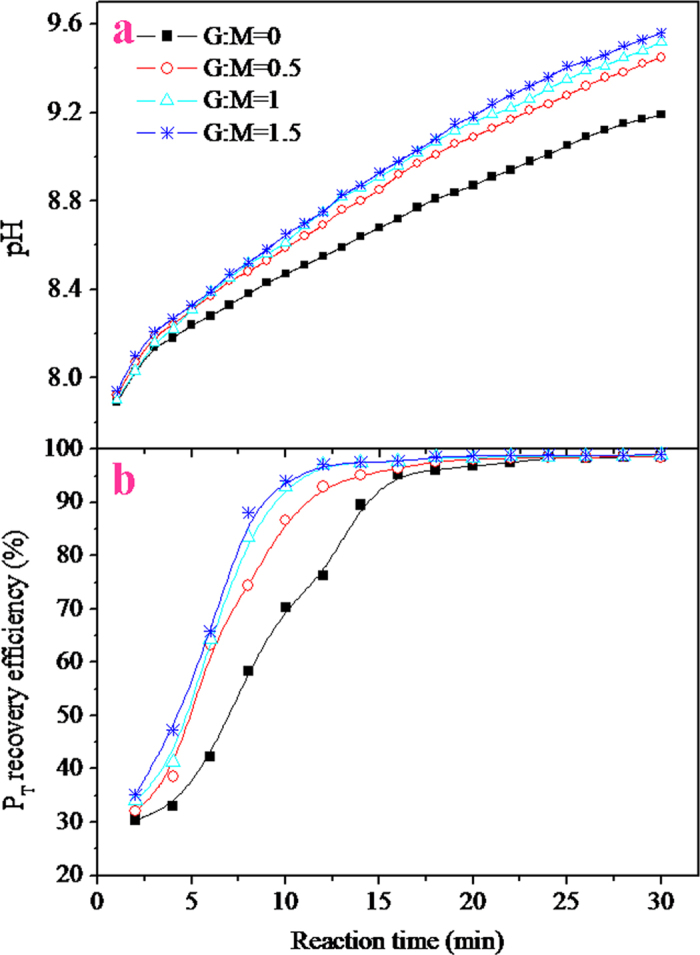
Effect of the G:M mass ratio on the solution pH and the P_T_ recovery efficiency (Mg metal dosage, 10 g L^–1^; airflow rate, 1 L min^–1^).

The effect of the G:M mass ratio on the struvite crystallization by the Mg metal corrosion is described in Fig. 4. It was found that as the G:M mass ratio increased the pH of the solution obviously increased (Fig. 4a). This may be attributed to the fact that while maintaining a constant dosage of the Mg metal, an increase in the G:M mass ratio resulted in an increase in the graphite dosage, which could increase the opportunity of the Mg metal to come into contact with the graphite pellets, causing the rise in the amount of the magnesium-graphite galvanic cell formed in the solution. Consequently, this accelerated the corrosion of the Mg metal, resulting in the increase in the solution pH. Nevertheless, when the G:M mass ratio was greater than 1:1, further increases in the G:M mass ratio caused a slight increase in the solution pH. This may be due to the amount of graphite used to form the magnesium-graphite galvanic cell tending to become sufficient. Correspondingly, the P_T_ recovery efficiencies also presented a similar changing tendency in response to the solution pH (Fig. 4b). Therefore, a 1:1 G:M mass ratio was adopted for the subsequent experiments.

#### Effect of airflow rate

**Figure 5:**
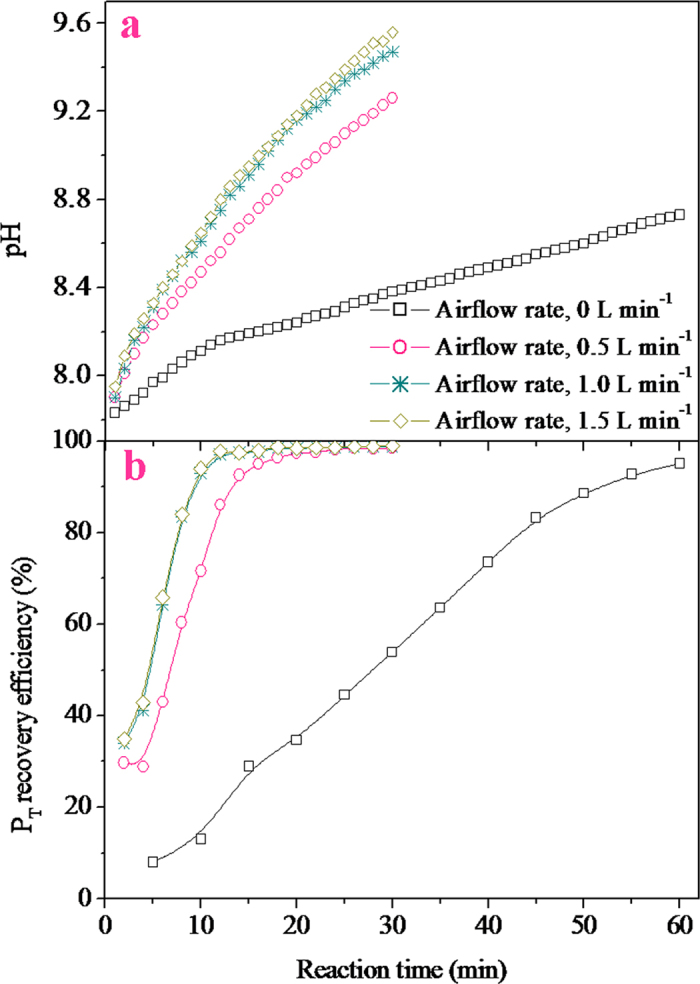
Effect of the airflow rate on the solution pH and the P_T_ recovery efficiency (G:M mass ratio, 1:1; Mg metal dosage, 10 g L^–1^).

The experimental results obtained for the struvite crystallization by Mg metal corrosion at different airflow rates are shown in Fig. 5, which reveal that the airflow rate significantly influenced the solution pH and the P_T_ recovery efficiency. As observed in Fig. 5a, when the graphite-magnesium corrosion system was not aerated, the solution pH increased very slowly to reach 8.73 over 60 min. However, when the aeration was at an airflow rate of 0.5 L min^–1^, the solution pH increased rapidly reaching a value of 9.26 for 30 min. Further increase in the airflow rate ranging from 0.5 L min^–1^ to 1 L min^–1^ could result in an additional increase in the solution pH; however, increasing the airflow rate over 1 L min^–1^ did not cause any further increase in the solution pH. As explained prior, the increase in the solution pH was mainly attributed to the solubility of the Mg(OH)_2_ film resulting from the corrosion of the Mg metal. Therefore, air bubbling could facilitate the stripping of the hydrogen gas formed and the Mg(OH)_2_ film from the Mg metal pellet surfaces, accelerating the corrosion of the Mg metal. In Fig. 5b, the changing profile of the P_T_ recovery efficiencies was observed to be similar to that of the solution pH. Based on economic considerations, it was confirmed that an airflow rate of 1 L min^–1^ was optimal for struvite formation by Mg metal corrosion. In the literature, a few papers dealing with the provocation of struvite crystallization by air bubbling technique^32^,^33^ were available, in which the struvite precipitation occurred after degassing the CO_2_ through aeration. In the investigations published, the degasification of CO_2_ by air bubbling could increase the solution pH, but a high airflow rate was normally necessary in the gas-liquid mass transfer process. In this study, as the air bubbling only served the graphite-magnesium system, a very small amount of air was required for the stripping process.

### Treatment of actual swine wastewater by the magnesium dosage process

#### P_T_ recovery by the multiple uses of the Mg metal pellets

**Figure 6:**
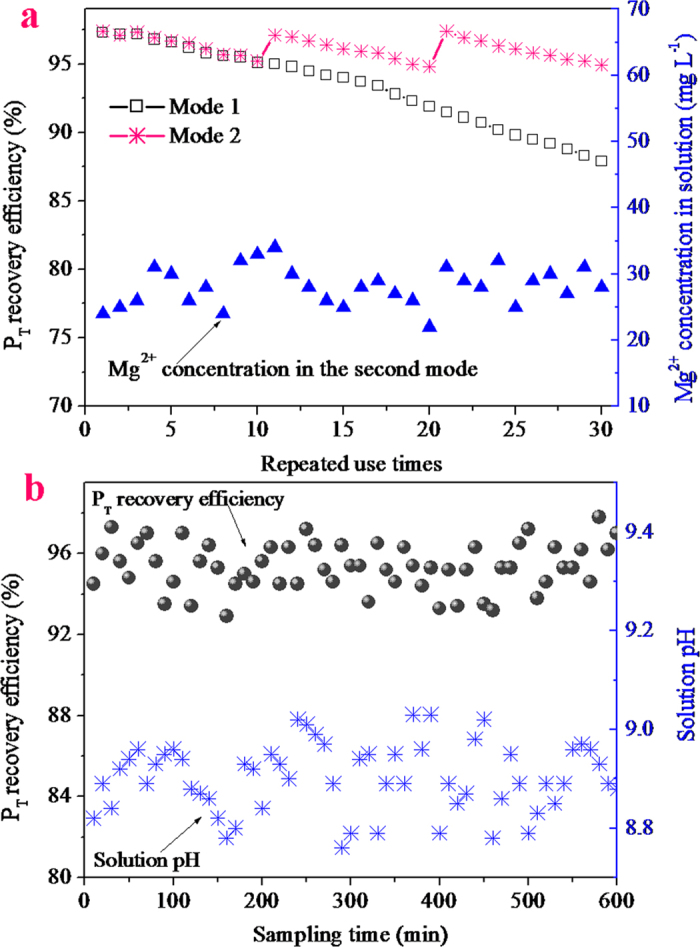
(**a**) the P_T_ recovery efficiencies and the remaining Mg^2+^ concentrations in the struvite precipitation by the corrosion of Mg metal at different repeated use times; (**b**) the P_T_ recovery efficiencies and the solution pHs in the continuous-flow reactor at different times.

Based on the operational procedures and the optimal conditions mentioned above (i.e. initial magnesium dosage, 10 g L^–1^; reaction time, 15 min; G:M mass ratio, 1:1; airflow rate, 1 L min^–1^), the graphite-magnesium air bubbling column was repeatedly used to recover P_T_ from actual swine wastewater for 30 cycles. In the experiments, two operational modes were investigated. In the first mode, the repeated use of the graphite-magnesium air bubbling column was performed without the supplementation of the Mg metal pellets. In the second mode, 0.5 g of Mg metal pellets was supplemented to the air bubbling column per 10 cycle uses (the Mg metal dosage was usually maintained at around 10 g L^–1^). The experimental results are shown in Fig. 6a, indicating that when the graphite-magnesium air bubbling column was constantly used without Mg metal supplementation, the P_T_ recovery efficiency was initially 97.3% and gradually decreased to 87.9% in the 30^th^ cycle of use. The P_T_ recovery efficiency decline could be explained as follows: With the increase in the use times, the Mg metal pellets were progressively sacrificed for the struvite crystallization and the residual Mg^2+^ in the supernatant per use cycle (Fig. 6a shows that the Mg^2+^ concentration of the supernatant in the second mode was maintained at around 28 mg L^–1^ during the experiments), which resulted in the decline in the mass and the surface area of the Mg metal pellets. Therefore, this resulted in a gradual decrease in the solution pH at the set time. When supplementing the Mg metal pellet to the air bubbling column, the P_T_ recovery efficiency was rapidly recovered to the initial value, and then gradually decreased with the increase in the repeated use times, similar to the stages in the first mode.

#### Continuous recovery of the phosphate from swine wastewater

**Figure 7:**
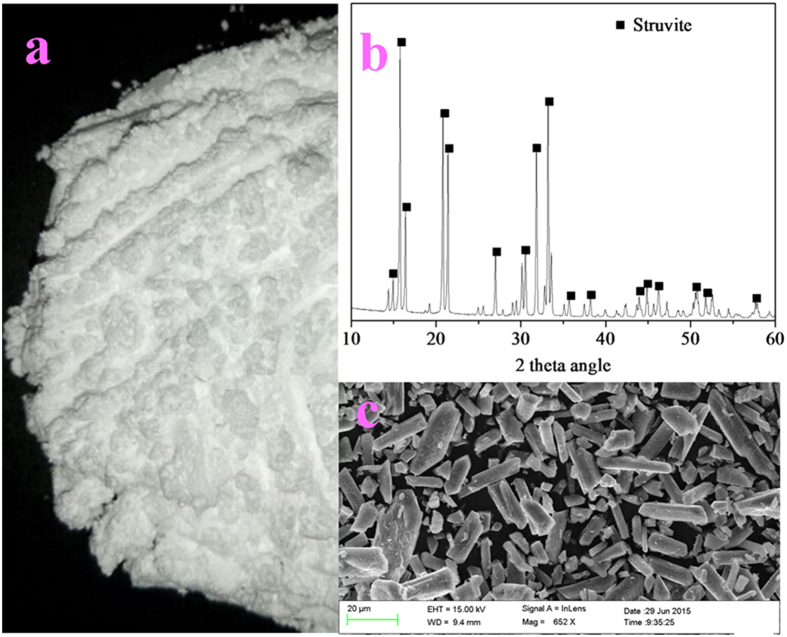
The picture (a), XRD patterns (b) and SEM micrograph (c) of the struvite collected from actual swine wastewater.

To facilitate operations, a continuous-flow reactor installed with the graphite-magnesium air bubbling column was used to recover the phosphate from the swine wastewater. In the lab-scale continuous-flow experiments, the operational conditions of the reaction were identical to those of the sequential batch experiments in the former Section (*P*_*T*_ *recovery by the multiple uses of the Mg metal pellets*). The difference between the two lay in the fact that in the continuous-flow experiments, the swine wastewater was continuously added to the reactor at a rate of around 66 ml min^–1^; in other words, the hydraulic retention time (HRT) of the reactor was around 15 min. To maintain the P_T_ recovery efficiency at a stable rate, approximately 0.5 g of Mg metal pellets was supplemented to the reactor at every 1 h intervals. Fig. 6b shows the P_T_ recovery efficiencies and the pH of the solution of the samples drawn at different times. This figure implies that during the experiments, the solution pH was basically maintained in the range of 8.8–9.0, which was the optimal pH range determined above. Under this pH condition, the P_T_ recovery efficiency was found to be maintained at around 95%. This suggested that recovering the phosphate from swine wastewater by the proposed continuous-flow reactor was completely feasible. Besides, the struvite precipitates collected from the precipitation tank (Fig. 7a) were dried and then characterized by SEM and XRD. The XRD diffractogram (Fig. 7b) indicates that the patterns of the recovered struvite concurred closely with the standard patterns of struvite (JCPDS-71-2089). The SEM picture (Fig. 7c) shows that the morphology of the struvite recovered from the actual swine wastewater by the continuous-flow reactor was identical to that collected from the synthetic swine wastewater. Furthermore, the composition of the recovered struvite precipitates was analyzed after dissolution using 0.10 M HNO_3_. The results demonstrated that the element contents of Mg, N, P, Ca and K in the recovered struvite were 97.5 ± 0.7, 55.7 ± 0.6, 125.9 ± 0.8, 8.7 ± 0.8 and 3.1 ± 0.3 mg/g, respectively, and the purity was 95.8% (±0.5).

In the published literatures available, some continuous-flow reactors have been developed for the phosphate recovery from the wastewaters^34^,^35^,^36^. Shepherd *et al.*^34^ reported a lab-scale continuous-flow reactor used to recover phosphate from swine manure slurries by air stripping for an increase in the pH and the mixing of the solution. This reactor could harvest phosphate of >93% from wastewater with the addition of MgCl_2_. However, the struvite crystals thus harvested cannot be separated from the reactor in a continuous-flow mode. Suzuki *et al.*^35^ also designed a continuous-flow reactor which could simultaneously induce the struvite crystallization by air stripping and separate the formed struvite by precipitation. Unfortunately, the total HRT for the reactor was too time consuming (22.3 h), and the recovery efficiency of the phosphate was relatively low (72.8%). Song *et al.*^36^ employed a continuous-flow reactor equipped with struvite accumulation devices for the recovery of phosphate from swine wastewater, and achieved a phosphate recovery of 85.4% in the reactor at a minimum HRT of 6.0 h. When compared with these reported literatures, we can see that the reactor proposed in this study shows many technical advantages such as the high potential for phosphate recovery (97.5%), low HRT (15 min) and high automation of the operation process.

### Cost analysis

As the total cost for the recovery of phosphate from swine wastewater mainly depends on the individual cost of the magnesium^37^, in this assessment, the costs incurred for the other aspects such as installation, maintenance, manpower and energy were not considered; the chemical cost of the magnesium alone was considered. According to the mass change of the Mg metal before and after the continuous-flow experiments mentioned above, the mass of the Mg metal consumed in the struvite crystallization process and the residual Mg^2+^ in the supernatant was statistically calculated to be around 100 mg per 1 L of swine wastewater. Therefore, considering the market price of pure Mg metal in 2015, the chemical cost of phosphate recovery by the continuous-flow reactor could be estimated at 0.25 $ kg^–1^ struvite or 0.21 $ m^–3^ swine wastewater (Table 1). In published literatures, there are a variety of processes dealing with the recovery of phosphate from waste^17^,^28^,^36^,^37^. The cost of phosphate recovery by these processes can not be well compared due to the difference in the operational conditions. Consequently, in order to further evaluate the economical feasibility of the proposed process, under the similar operational conditions, the cost for dosing the magnesium by the corrosion of the Mg metal was only compared with those for struvite precipitation by dosing the frequently used chemical reagents containing magnesium (i.e. MgO, MgCl_2_ and MgSO_4_). As shown in Table 1, it was found that the cost for dosing the magnesium by the corrosion of Mg metal was higher than that of using the MgO, but was comparable with those of using MgCl_2_ and MgSO_4_. The findings regarding the cost of the magnesium dosage by the corrosion of the Mg metal was consistent with that of the electrochemical magnesium dosage reported by Hug and Udert^17^. This suggested that the dosing magnesium for the struvite crystallization by the corrosion of Mg metal is competitive with the dosage of most magnesium salts in the economy. Additionally, on comparing the uses of the magnesium salts and MgO/Mg(OH)_2_, the proposed magnesium dosage process involves simpler handling and is not troublesome, as mentioned earlier. Therefore, this process system would be particularly interesting for the industrial recovery of phosphate because the reactor is easy to automatically operate and is scalable in size.

## Conclusions

Phosphate in swine wastewater can be harvested efficiently through struvite crystallization by the corrosion of Mg metal. Dosing magnesium using the graphite-magnesium air bubbling column was the optimal operational mode. The optimal conditions for the process were found to involve Mg metal dosage 10 g L^–1^, G:M mass ratio 1:1 and airflow rate 1 L min^–1^, with a P_T_ recovery efficiency of 97.5%. A continuous-flow reactor using the proposed magnesium dosage process was developed for phosphate recovery from actual swine wastewater and could achieve a stable P_T_ recovery efficiency (around 95%). The cost analysis demonstrated that the process proposed was economically feasible.

## Materials and Methods

### Materials

Swine wastewater from a pig farm located at the suburb area of Beijing was used for the experiments, after screening via filter paper. Its specific components are shown in Table 2. Besides referring to the wastewater characteristics, synthetic swine wastewater with the same P_T_ and ammonia nitrogen (NH_3_-N) concentrations to the actual wastewater was prepared by dissolving analytical grade NH_4_Cl and Na_2_HPO_4_·12H_2_O in deionized water. Before use, the pH of the synthetic swine wastewater was adjusted to 7.8 using 0.1 M NaOH. The Mg metal pellets as an Mg^2+^ source and maintenance of alkalinity for the crystallization of struvite were purchased from Tianjing Yanhong Nano Technology Co., Ltd. The particle size and purity were 2–3 mm and 99.9%, respectively. Some graphite pellets of particle size and carbon content of 0.3–0.5 mm and 99.5%, respectively, were used to accelerate the corrosion of Mg metal, supplied by the Changsha Shande Graphite Plant.

### Reactor setup and experimental methods

**Figure 8:**
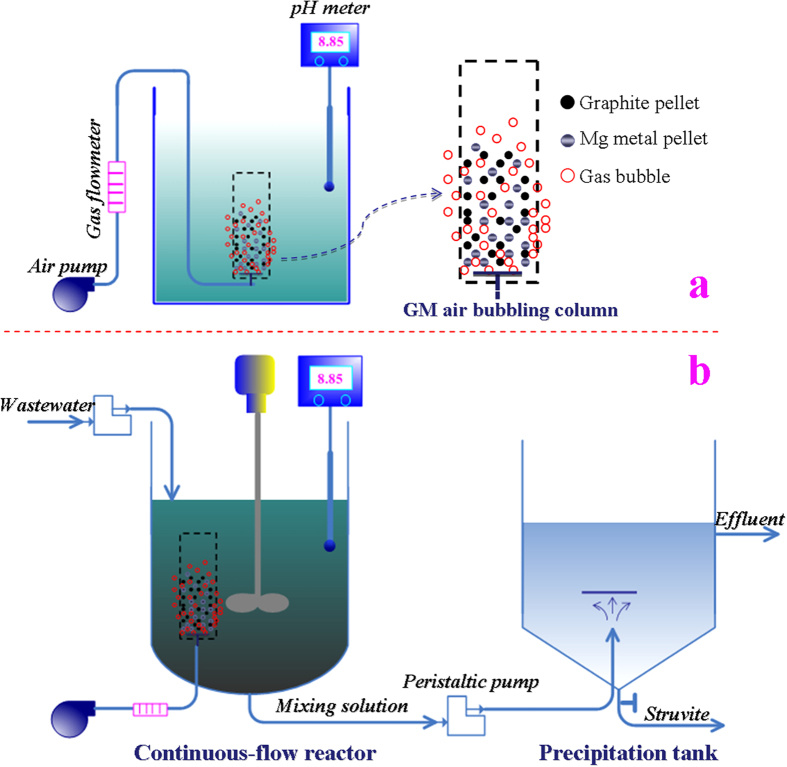
Schematic illustrations of (a) the batch reactor and (b) the continuous-flow reactor using the graphite-magnesium air bubbling column.

To determine the mechanism and conditions of the struvite crystallization process via the corrosion of Mg metal, a series of batches of experiment were performed in a single-compartment reactor with a maximum liquid volume of 1 L, in which synthetic swine wastewater was used as the working solution. The schematic representation of the batch reactor is shown in Fig. 8a. An air bubbling column made of plastic net, pore size 0.1 mm was immersed into the reactor and used to load the Mg metal and graphite pellets. A coarse air bubble diffuser was mounted at the bottom of the column to provide diffused aeration for the fluidization of the Mg metal and graphite pellets. In the experiments, the struvite crystallization via the corrosion of Mg metal was first investigated at four different operational modes. The basic operational procedures involved in the four modes are as follows: 500 ml of synthetic swine wastewater was first poured into the reactor, and then the required Mg metal pellets (3g) was added to the solution, and rapidly stirred at 300 rpm. The pH of solution was recorded at different times by a pH meter, and 1 mL of the solution at different intervals was removed and filtered through a 0.2-μm filter membrane for component analysis. The differences of the four modes were described as follows. In the first operational mode (*M*_1_), the Mg metal pellets was directly added to the solution; whereas in the second operational mode (*M*_2_), besides the Mg metal pellets, 3g of graphite pellets was additionally added to the solution (the mass ratio of graphite and magnesium was 1:1, i.e. G:M = 1:1). In the third mode (*M*_3_), the Mg metal pellets was added into the air bubbling column, and bubbled at an airflow rate of 1 L min^–1^; whereas in the fourth operational mode (*M*_4_), the Mg metal pellets plus 3g of graphite pellets were added to the air bubbling column, and aeration was performed at an airflow rate of 1 L min^–1^. Based on the optimal operational mode, the conditional experiments were conducted to obtain the optimal parameters of the Mg metal dosage, G:M mass ratio and airflow rate for the P_T_ recovery from swine wastewater.

Besides, a continuous-flow reactor was developed to facilitate the operation of the proposed P_T_ recovery process. As shown in Fig. 8b, the continuous-flow reactor was comprised of a barrel with a conical bottom, and had a reacting zone of 1 L. A mechanical agitator and the designed air bubbling column were installed at the tip and bottom of the reactor, respectively. The mixing solution in the reactor was continuously pumped into the precipitation tank to enable the separation of struvite solids. The precipitated struvite was removed from the tank bottom. During the continuous-flow experiments, at different time intervals, 5 ml samples were taken from the supernatant of the precipitation tank to determine the efficiency of the P_T_ recovery.

### Analytical methods

The components of the screened swine wastewater were analyzed according to the standard methods^38^. After the required pretreatment of the samples, the concentrations of NH_4_-N and P_T_ were determined by the Nessler’s reagent spectrophotometric method and Mo–Sb anti-spectrophotometric method (752 N-spectrophotometer; China), respectively. The concentrations of the cations like Ca^2+^, K^+^, Mg^2+^, Fe^2+^/Fe^3+^, Zn^2+^ and Cu^2+^ were measured by an atomic adsorption photometer (AA-6800; Shimadzu, Japan). The solution pH was measured by a pH meter (pHS-3C; China). The struvite precipitates collected during the experiments were washed thrice with pure water, and then oven dried at 35 °C for 48 h. The morphology of the dried struvite solids was observed using a scanning electron microscope-energy dispersive spectrometer (SEM-EDS; SUPRA 55 SAPPHIRE; Germany), and the composition was analyzed using an X-ray diffraction analyzer (XRD; DMAX-RB; Rigaku, Japan).

## Additional Information

**How to cite this article**: Huang, H. *et al.* Crystallization and precipitation of phosphate from swine wastewater by magnesium metal corrosion. *Sci. Rep.* **5**, 16601; doi: 10.1038/srep16601 (2015).

## Figures and Tables

**Table 1:** The market prices of the various chemicals and cost analysis of various chemicals used as magnesium sources.

Chemicals	Marketprice	Costs for using differentmagnesium reagents($ kg^–1^ struvite)			
	($ kg^−1^)	Mg metal	MgO^a^	MgCl_2_^b^	MgSO_4_^b^
Mg metal (99.9%)	2.1	0.25	—	—	—
MgO (95%)	0.12	—	0.05	—	—
MgCl_2_·6H_2_O	0.12	—	—	0.12	—
MgSO_4_·7H_2_O	0.13	—	—	—	0.17
NaOH	0.46	—	—	0.11	0.11
Total	—	0.25	0.05	0.23	0.28

^a^Dosing MgO at a Mg:P molar ratio of 2.5:1.

^b^Dosing MgCl_2_/MgSO_4_ at a Mg:P molar ratio of 1.2:1, pH = 9.

**Table 2:** The characteristics of the screened swine wastewater used in the experiments.

Parameter	Average values plusStandard deviation
pH	7.8 ± 0.06
COD (mg L^–1^)	3901 ± 215
BOD_5_ (mg L^–1^)	2052 ± 332
Alkalinity (as Na_2_CO_3_) (mg L^–1^)	3211 ± 253
P_T_ (total orthophosphate, mg L^–1^)	112 ± 6.2
NH_4_-N (ammonia nitrogen, mg L^–1^)	677 ± 32
Cl^–^(mg L^–1^)	1352 ± 121
K (mg L^–1^)	252 ± 22
Ca (mg L^–1^)	42 ± 4.5
Mg (mg L^–1^)	14 ± 1.8
Fe (mg L^–1^)	1.2 ± 0.6
Cu (mg L^–1^)	0.5 ± 0.1
Zn (mg L^–1^)	0.7 ± 0.1
